# Positive association between advanced lung cancer inflammation index and gallstones, with greater impact on women: a cross-sectional study of the NHANES database

**DOI:** 10.3389/fnut.2024.1506477

**Published:** 2024-12-20

**Authors:** Bailiang Liu, Luyuan Jin, Boyuan Nan, Zhongyi Sun, Fengyang Chen, Yinghui Zhou, Qila Sa, Yingnan Feng, Ao Men, Wenxin Wang, Xiaodong Feng, Wei Zhang

**Affiliations:** ^1^Department of Hepatobiliary Surgery, General Hospital of Northern Theater Command, Shenyang, China; ^2^Postgraduate College, Dalian Medical University, Dalian, China

**Keywords:** advanced lung cancer inflammation index, gallstones, NHANES, inflammation, nutrition, cross-sectional study

## Abstract

**Background:**

Previous studies have shown that inflammation is crucial in gallstone formation. The Advanced Lung Cancer Inflammation Index (ALI) is a comprehensive measure that reflects inflammation and nutritional condition. However, there are no studies examining the relationship between ALI and gallstones. This study aimed to analyze this association in US adults.

**Methods:**

This study used a cross-sectional research design with in-depth analyses using data from the National Health and Nutrition Examination Survey (NHANES). The association between gallstones and ALI was systematically assessed by logistic regression analysis, subgroup analysis, basic participant characteristics, and smooth curve fits.

**Results:**

5,646 people participated in the study. ALI was converted into Quartile 1 (−1.47–1.00), Quartile 2 (1.00–1.34), Quartile 3 (1.34–1.69), and Quartile 4 (1.69–4.38). In the fully adjusted model, gallstone prevalence increased by 45% in participants in the highest quartile compared to those in the lowest quartile (OR = 1.45; 95% CI: 1.12–1.87; *p* = 0.005), and ALI was positively correlated with gallstones (OR = 1.22; 95% CI: 1.03–1.45; *p* = 0.0232). Smooth curve fits provided evidence in favor of this finding. Significant gender differences were found in the relationship between gallstones and ALI by subgroup analysis (OR = 1.43; 95% CI: 1.16–1.76; *p* for interaction = 0.0204).

**Conclusion:**

The study concluded that ALI and gallstones had a positive correlation, with ALI having a higher effect on women’s gallstone prevalence.

## 1 Introduction

Gallstone disease is a common digestive system disorder and has become an important issue in modern healthcare. With an annual incidence of 0.60–1.3% (1), the prevalence of gallstones in US adults hovers around 10–20%, and this number is increasing (2). Although gallstones are usually asymptomatic, they can still be harmful. More than 20% of gallstone patients eventually experience common symptoms including colic or infection (3). A few patients may even develop rare complications such as gallstone ileus, Mirizzi syndrome, and Bouveret syndrome (4, 5). These symptoms cause great discomfort to the patient and impose an enormous financial strain on society and families. Therefore, gallstones are considered a severe public health concern that deserves vigilant attention from the medical community.

It is generally recognized that inflammation contributes significantly to the many factors that lead to gallstone development (6–8). Several inflammatory markers have been identified as potential indicators of the promotion of gallstone formation. C reactive protein and high-sensitivity C reactive protein, indicators of systemic inflammation, have been linked to an elevated risk of gallstone disease (9, 10). Interleukin (IL)-6, a kind of cytokine, has been linked to gallstone formation due to its role in promoting inflammation (8). Additionally, there is clear evidence linking the systemic immune-inflammatory index to a significant increase in gallstone prevalence (11).

Integrating BMI, albumin, and neutrophil to lymphocyte ratio (NLR), the advanced lung cancer inflammation index (ALI) is a systemic inflammatory index. ALI was initially evaluated for lung cancer patients (12). Given the value of the inflammatory aspects of the ALI assessment, several studies have explored its role in hypertension, diabetes, heart failure, rheumatoid arthritis, coronary artery disease, and stroke (13–18). Although inflammation is known to be linked to gallstones, it is unclear how ALI and gallstones are related.

Therefore, we aimed to use National Health and Nutrition Examination Survey (NHANES) to explore the association between ALI and gallstones among US adults.

## 2 Materials and methods

### 2.1 Study population

NHANES is a crucial resource offering extensive health and nutrition data from a nationally representative sample of the US population. It covers a wide array of health indicators and diseases through interviews and physical examinations, enabling researchers to identify risk factors, prevalence rates, and trends in public health. This comprehensive dataset supports epidemiological research and informs public health policy decisions, making it an invaluable tool for understanding the health status of Americans.

**Figure 1:**
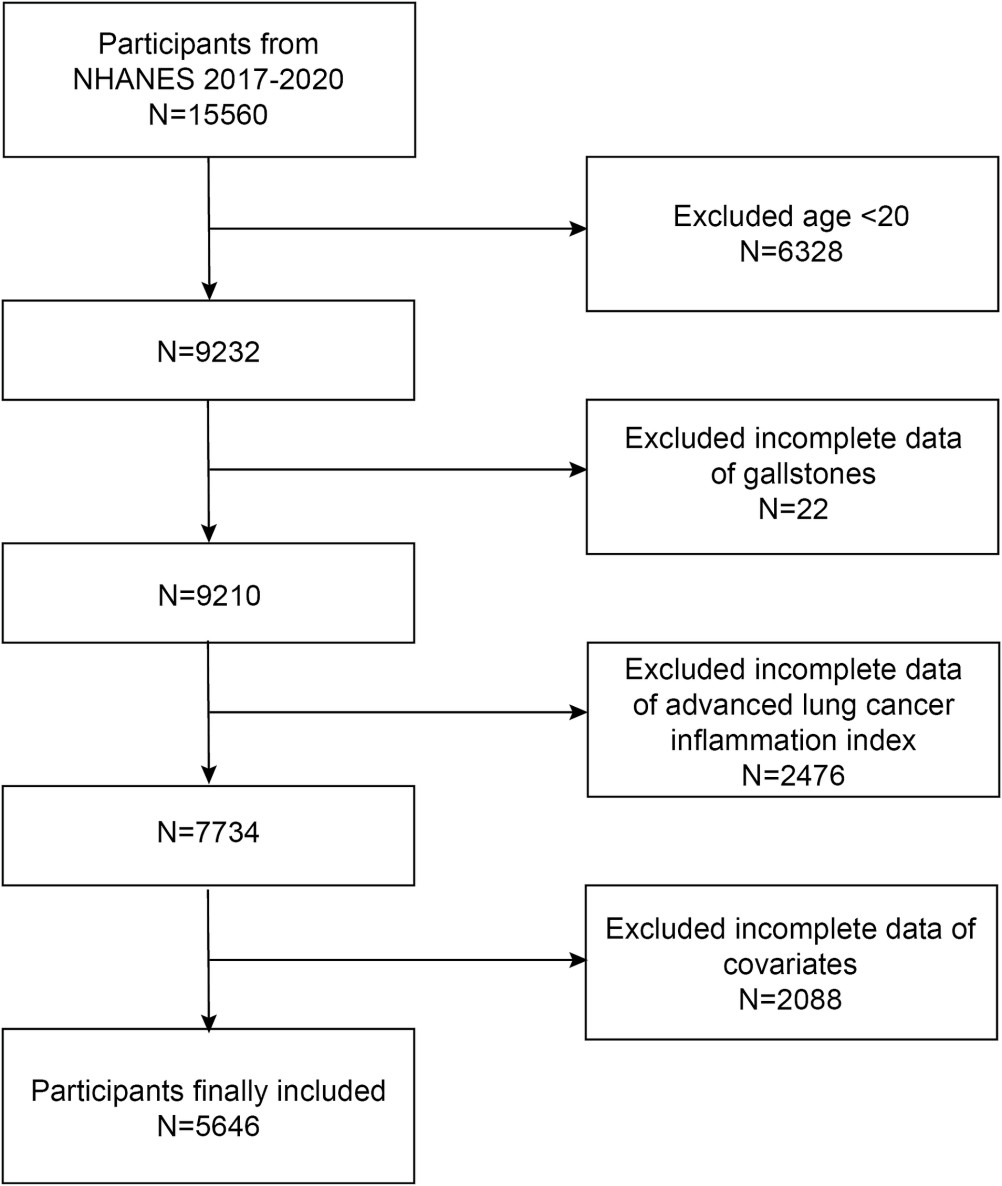
Sample selection criteria flowchart.

We used NHANES data from 2017 through March 2020 for our analysis. There were initially 15,560 participants, of which 6,328 participants younger than 20 were excluded. Participants lacking information about gallstones were excluded (*n* = 20). Participants lacking ALI were excluded (*n* = 2,476). Additionally, participants lacking data on covariates were excluded [total, *n* = 2088; including education level, *n* = 9; marital status, *n* = 5; alcohol consumption, *n* = 1,074; smoking status, *n* = 4; diabetes, *n* = 201; hypertension, *n* = 7; poverty-to-income ratio (PIR), *n* = 788]. In the end, there were 5,646 individuals in total for the research (Figure 1).

### 2.2 Definition of gallstones

Based on the answers to the question “Has DR ever said you have gallstones?” we were able to assess whether or not the individuals had gallstones. Gallstones were classified as present in those who answered “yes,” whereas they were not in those who answered “no”.

### 2.3 Measurement of ALI

ALI was computed using the formula BMI (kg/m^2^)*albumin level (g/dL)/NLR. NLR was computed by dividing neutrophil counts by lymphocyte counts.

### 2.4 Covariables

Our investigation took into account additional confounding factors that could alter the relationship between ALI and gallstones. Based on previously published literature (11, 19–21), the covariates chosen were age, gender, race, educational level, PIR, marital status (cohabitation/solitude), alcohol consumption, smoking status, diabetes, hypertension, BMI, albumin, neutrophil counts, and lymphocyte counts. Cohabitation in marital status included married and living with partner, and solitude included widowed, divorced, separated, and never married. Those who had ever smoked 100 cigarettes or more were considered smokers. Drinkers were those participants who consumed alcohol at least once a month. Diabetes status was determined by participants’ answers to the question “Doctor told you have diabetes”; and a similar questionnaire was used to identify hypertension.

### 2.5 Statistical analysis

The study was statistically analyzed using R (version 3.4.3) and EmpowerStats (version 2.0). Statistical significance is indicated by a *p* value of less than 0.05. Two groups of participants were created based on whether or not gallstones were present. The ALI was subjected to a ln transformation because of its skewed distribution, which was then analyzed as a continuous variable. Four groups of participants were formed based on the quartiles of the ALI. Continuous variables were tested using Student’s t-test and categorical variables using chi-square test. In three distinct models, multivariate logistic regression methods were utilized in the investigation of the association between the prevalence of gallstones and ALI. No covariates were adjusted in model 1. Age, race, and gender were adjusted in model 2. Model 3 took into account factors such as gender, age, race, education level, PIR, marital status, alcohol consumption, smoking, diabetes, and hypertension. Subgroup analysis were conducted using logistic regression models. Furthermore, we evaluated the nonlinear relationship between the prevalence of gallstones and ALI using smooth curve fits.

## 3 Results

### 3.1 Baseline characteristics

The 5,646 individuals’ initial characteristics are displayed in Table 1. The average age was 50.43 ± 17.33 years, with 50.21% male and 49.79% female individuals. The prevalence of gallstones was 10.63%. There were significant differences between the groups with and without gallstones in age, gender, race, alcohol consumption, smoking status, diabetes, hypertension, BMI, albumin, neutrophil counts, and lymphocyte counts. However, ALI between gallstone formers and non-gallstone formers was not significantly different (*p* = 0.5080). Compared to non-gallstones participants, gallstone participants were more likely to be older, female, predominantly other Hispanic, smokers, non-drinkers, and to have diabetes, hypertension, higher BMI, higher albumin levels, higher neutrophil counts, and higher lymphocyte counts.

**Table 1:** Baseline characteristics of participants.

Characteristics	Non-stone formers (*n* = 5,046)	Stone formers (*n* = 600)	*p*-value
Age (years)	49.58 ± 17.35	57.53 ± 15.49	<0.001
Gender, *n* (%)			<0.001
Male	2,663 (52.77)	172 (28.67)	
Female	2,383 (47.23)	428 (71.33)	
Race, *n* (%)			<0.001
Mexican American	585 (11.59)	82 (13.67)	
Other Hispanic	481 (9.53)	69 (11.50)	
Non-Hispanic White	1956 (38.76)	275 (45.83)	
Non-Hispanic Black	1,296 (25.68)	107 (17.83)	
Other race	728 (14.43)	67 (11.17)	
Education level, *n* (%)			0.505
Less than high school	786 (15.58)	101 (16.83)	
High school or GED	1,211 (24.00)	151 (25.17)	
More than high school	3,049 (60.42)	348 (58.00)	
PIR	2.66 ± 1.63	2.61 ± 1.58	0.429
Marital status, *n* (%)			0.098
Cohabitation	2,934 (58.15)	370 (61.67)	
Solitude	2,112 (41.85)	230 (38.33)	
Alcohol consumption, *n* (%)			<0.001
Yes	2,703 (53.57)	238 (39.67)	
No	2,343 (46.43)	362 (60.33)	
Smoking status, *n* (%)			0.001
Yes	2,259 (44.77)	311 (51.83)	
No	2,787 (55.23)	289 (48.17)	
Diabetes, *n* (%)			<0.001
Yes	720 (14.27)	161 (26.83)	
No	4,326 (85.73)	439 (73.17)	
Hypertension, *n* (%)			<0.001
Yes	1816 (35.99)	318 (53.00)	
No	3,230 (64.01)	282 (47.00)	
ALI	72.36 ± 46.51	73.76 ± 64.40	0.508
BMI (kg/m^2^)	29.82 ± 7.38	33.46 ± 8.47	<0.001
Albumin (g/dL)	4.07 ± 0.34	3.95 ± 0.35	<0.001
Neutrophil counts (K/uL)	4.17 ± 1.69	4.48 ± 2.03	<0.001
Lymphocyte counts (K/uL)	2.20 ± 1.36	2.78 ± 14.58	0.007

The mean ± standard deviation (SD) was used to record continuous variables. Frequencies or percentages was used to express categorical variables. ALI, advanced lung cancer inflammation index; BMI, body mass index; PIR, poverty-to-income ratio; GED, general educational development.

### 3.2 Association between ALI and gallstones

**Figure 2:**
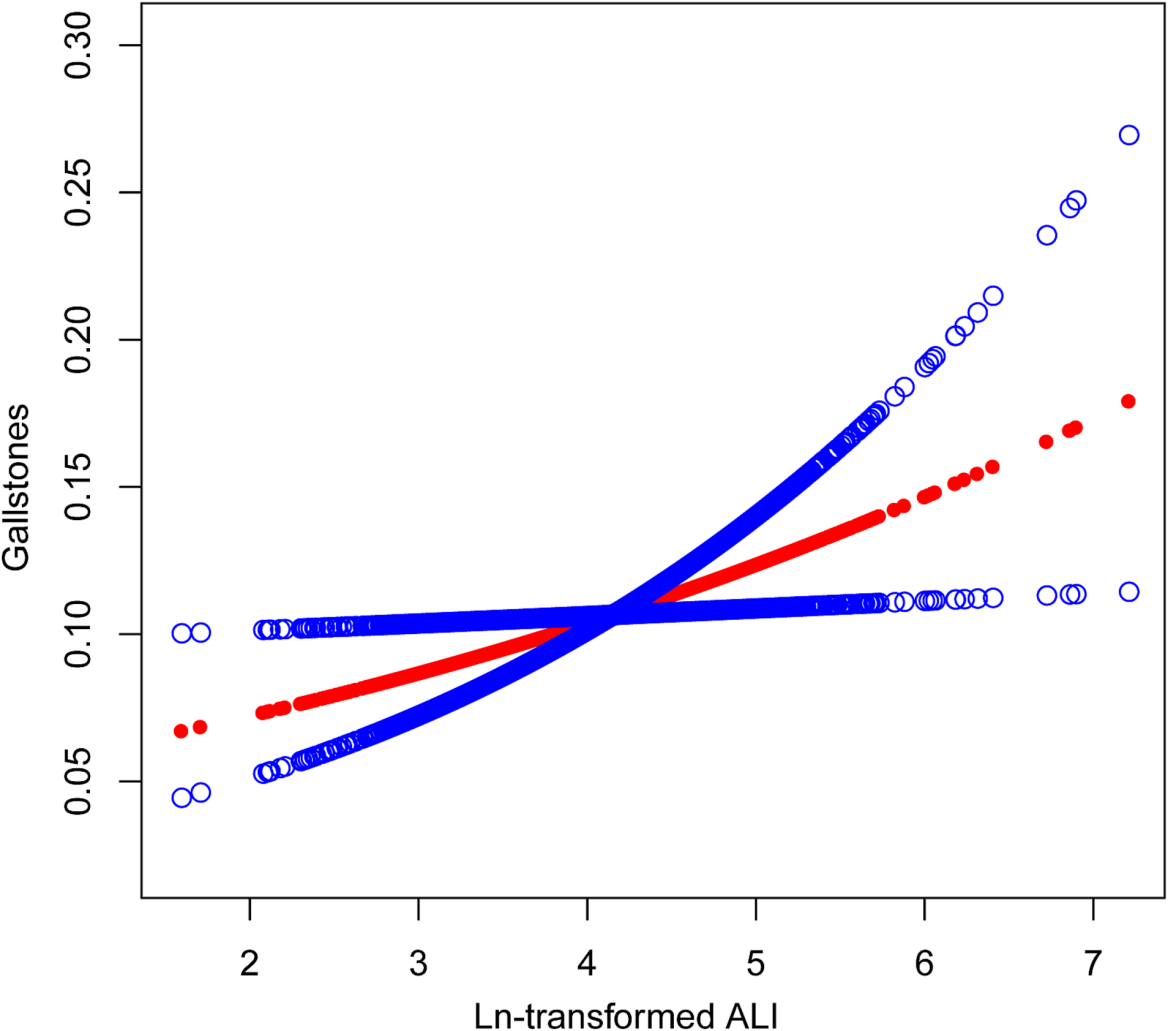
Linear relationship between ln-transformed ALI and gallstones.

Table 2 shows the association between ln-transformed ALI and gallstones. No correlation between ALI and gallstones was found in Model 1, which was not adjusted for confounding factors. However, after adjusting for confounding factors, a positive correlation between ALI and the prevalence of gallstones was detected in both Model 2 and Model 3. In Model 3, there was a 22% rise in gallstone prevalence for every unit rise in ln-transformed ALI (OR = 1.22; 95% CI: 1.03–1.45; *p* = 0.0232). We calculated a cut-off value of 4.39 for ln-transformed ALI, above which the risk of developing gallstones increases. Then we convert the continuous ln-transformed ALI to Quartile 1 (−1.47–1.00), Quartile 2 (1.00–1.34), Quartile 3 (1.34–1.69), and Quartile 4 (1.69–4.38). We still get the same results after converting ALI to quartiles for analysis. Gallstone prevalence was 45% higher in Quartile 4 participants than it was in Quartile 1 participants (OR = 1.45; 95% CI: 1.12–1.87; *p* = 0.005). The *p* for trend analysis similarly supports the above findings in Model 2 (*p* = 0.0046) and Model 3 (*p* = 0.01). The results of the smooth curve fits provided additional evidence for the positive association between gallstones prevalence and ALI (Figure 2).

**Table 2:** The association between ln-transformed ALI and gallstones.

Characteristic	OR (95% CI), *p* value		
	Model 1	Model 2	Model 3
Ln-transformed ALI (continuous)	1.03 (0.88, 1.21) 0.7241	1.23 (1.04, 1.46) 0.0161	1.22 (1.03, 1.45) 0.0232
Ln-transformed ALI (quartile)			
Quartile 1 (−1.47–1.00)	Reference	Reference	Reference
Quartile 2 (1.00–1.34)	1.07 (0.84, 1.36) 0.5776	1.21 (0.95, 1.56) 0.1246	1.21 (0.94, 1.56) 0.1329
Quartile 3 (1.34–1.69)	0.96 (0.75, 1.23) 0.7596	1.15 (0.89, 1.49) 0.2746	1.14 (0.88, 1.48) 0.3113
Quartile 4 (1.69–4.38)	1.12 (0.89, 1.43) 0.3329	1.49 (1.15, 1.92) 0.0022	1.45 (1.12, 1.87) 0.0050
P for trend	0.4762	0.0046	0.0100

In sensitivity analysis, ln-transformed ALI was converted from a continuous variable to a categorical variable (quartiles). ALI, advanced lung cancer inflammation index; OR, odds ratio; 95% CI, 95% confidence interval. Model 1: no covariates were adjusted. Model 2: age, gender, and race were adjusted. Model 3: age, gender, race, educational level, poverty-to-income ratio, marital status, alcohol consumption, smoking status, diabetes, and hypertension were adjusted.

### 3.3 Subgroup analysis

We used subgroup analysis and interaction tests to detect the association between ALI and gallstones in different subgroups. Gender, race, education level, marital status, alcohol consumption, smoking, diabetes, and hypertension were included in subgroup analysis as stratification factors (Table 3). The subgroup analysis’s findings demonstrated that, except for gender, none of the other stratification factors significantly interacted with the ALI and gallstones (p for interaction>0.05). Significant gender differences in the association between ALI and gallstones. A higher ALI was more strongly linked to women’s gallstone prevalence (OR = 1.43; 95% CI: 1.16–1.76; *p* for interaction = 0.0204). Thus, the alterations in the ALI had a greater impact on women’s gallstone prevalence.

**Table 3:** Association between ln-transformed ALI and gallstones in subgroups.

Subgroup	OR (95% CI)	*p* value	P for interaction
Gender			0.0204
Male	0.93 (0.69, 1.26)	0.6583	
Female	1.43 (1.16, 1.76)	0.0007	
Race			0.7734
Mexican American	1.15 (0.69, 1.94)	0.5924	
Other Hispanic	1.17 (0.68, 2.02)	0.5626	
Non-Hispanic White	1.27 (0.98, 1.64)	0.0655	
Non-Hispanic Black	0.99 (0.69, 1.43)	0.9753	
Other Race	1.46 (0.87, 2.47)	0.1560	
Education level			0.3527
Less than high school	0.91 (0.60, 1.40)	0.6722	
High school or GED	1.30 (0.91, 1.86)	0.1435	
More than high school	1.28 (1.02, 1.59)	0.0308	
Marital status			0.1504
Cohabitation	1.22 (0.98, 1.52)	0.0694	
Solitude	1.21 (0.93, 1.58)	0.1504	
Alcohol consumption			0.7484
Yes	1.20 (0.96, 1.49)	0.1053	
No	1.27 (0.96, 1.67)	0.0912	
Smoking status			0.6832
Yes	1.17 (0.93, 1.48)	0.1896	
No	1.25 (0.98, 1.60)	0.0670	
Diabetes			0.6882
Yes	1.29 (0.92, 1.81)	0.1385	
No	1.19 (0.98, 1.45)	0.0808	
Hypertension			0.8488
Yes	1.22 (0.97, 1.54)	0.0921	
No	1.18 (0.92, 1.51)	0.1836	

The results of subgroup analysis were adjusted for all covariates except the effect modifier. OR, odds ratio; 95% CI, 95% confidence interval; GED, general educational development.

## 4 Discussion

The relationship between ALI and gallstones among US adults is being examined for the first time with our cross-sectional investigation. The results suggested that elevated ALI was significantly associated with a higher gallstone prevalence after further adjustment for potential confounding variables. The smooth curve fits result further demonstrated the positive correlation. Additionally, the association between gallstones and ALI varied significantly according to gender. The prevalence of gallstones is more likely to be affected by high ALI levels in female individuals.

The impact of inflammation on gallstones is receiving more and more widespread attention. In a cross-sectional investigation involving 4,950 US adult participants based on the NHANES database, Meng et al. discovered that a higher systemic immune-inflammatory index was independently linked to the probability of gallstone prevalence under 50 (11). In a case–control research including 150 Iranian women, Ghorbani et al. discovered a considerable positive correlation between the incidence of gallstones and high-sensitivity C reactive protein (22). This is similar to our finding that the prevalence of gallstones in women is positively correlated with ALI levels. Pro-inflammatory diets can increase systemic levels of inflammation, and several studies have found that increased intake of pro-inflammatory foods is linked to an elevated risk of gallstones (19, 22). High levels of circulating inflammatory proteins, such as IL-6, IL-10, IL-12 (p70), and IL-13, were linked to an elevated incidence of gallstones, according to a cross-sectional study by Liu et al. with 299 participants from Shanghai, China (8). In a case–control study, Hsing et al. found that variants in several genes influencing inflammatory responses were associated with gallstones (23). Our research shows a correlation between high levels of ALI and higher gallstones prevalence, this agrees with the findings described above.

Gallstone development is influenced by environmental and genetic risk factors (3, 24). Among these risk factors, inflammation and nutritional status have a substantial effect on gallstone formation. Albumin, as one of the indicators of nutritional status, was found to be statistically significant in our study in the difference between gallstone formers (3.95 ± 0.35) and non-gallstone formers (4.07 ± 0.34). However, the difference is not clinically significant in clinical practice. The ALI is obtained from BMI, albumin, and NLR calculations and is an all-encompassing representation of the body’s nutritional and inflammatory conditions. Elevated BMI, one of the indicators used to assess obesity, is independently correlated with gallstone formation (25, 26). Our study found that the mean BMI of gallstone formers was 33 kg/m^2^, meaning they have obesity class I, which also supports the above conclusions. Obese state is an inflammatory state, which is also associated with gallstone formation. Obese patients usually have higher cholesterol and triglycerides (27). In turn, higher cholesterol and triglyceride levels are associated with an increased risk of gallstone disease (3). High levels of insulin in obese individuals can lead to increased cholesterol production by stimulating 3-hydroxy-3-methylglutaryl-coenzyme a reductase activity, which consequently raises gallstones risk (28, 29). Immune cells and multiple kinds of inflammatory cytokines are involved in gallstone formation. One study carried out on immunodeficient mice demonstrated a noteworthy rise in gallstone occurrence after the transfer of T lymphocytes (30). Neutrophils are more likely to trespass into the bile in an inflammatory state, and neutrophil extracellular traps formed by neutrophils and their extracellular DNA can promote gallstone formation by agglomerating calcium and cholesterol crystals (31). Our study found a significant gender difference in ALI and gallstones, with a more significant association in women. This may be related to gender differences in immune function. Compared to men, women have higher levels of estrogen in their bodies. The number of neutrophils is higher in high estrogen populations (32) and estrogens also modify cellular communication to enhance neutrophil activity (33). In turn, increased neutrophils can further promote gallstone formation through pathways such as neutrophil extracellular traps (31).

This study reveals a positive correlation between ALI and the prevalence of gallstones. Specifically, the prevalence of gallstones increased significantly when the ln-transformed ALI exceeded 4.39. This finding not only provides a new perspective for understanding the mechanism of gallstone formation, but also provides guidance for clinical practice. In clinical practice, special attention should be paid to screening and prevention of gallstones in patients with high ALI. Regular ultrasonography and other imaging tests are performed to aid in the early detection of gallstones, leading to timely intervention. We also found this relationship to be more pronounced in women. Therefore, in clinical practice, more attention should be paid to monitoring and controlling ALI in female patients, especially those with higher ALI, to reduce the risk of gallstones.

There are various advantages to this study. The relationship between ALI and gallstones is being examined for the first time in this study. The study is based on the NHANES database, which offers a more objective picture of the American people and has a bigger and more representative sample size. The study was adjusted for confounding covariates to guarantee more reliable findings. Additionally, in order to examine the strength of the correlation between gallstones and ALI in various groups, we conducted subgroup analyses. This study contains several restrictions. It was not possible to investigate a causal link between ALI and gallstones because the study was cross-sectional. Gallstone, diabetes, and hypertension diagnosis were based on questionnaires without objective exams, which made the diagnosis less objective. Although several covariates were considered in the study, we were unable to exclude the influence of all potential confounders such as genetics, hematological disorders, rapid weight loss, previous bypass surgeries, cholesterol, and triglycerides.

## 5 Conclusion

In this study, we found that ALI was positively associated with gallstones and that ALI had a higher effect on women’s gallstone prevalence. The findings suggest that early and effective management of ALI may reduce the prevalence of gallstones and have greater benefits for the female population. To validate the authors’ findings, larger prospective studies are still required.

## Data Availability

Publicly available datasets were analyzed in this study. This data can be found here: https://www.cdc.gov/nchs/nhanes/index.htm.
